# Heart Failure Secondary to Left Ventricular Non-compaction Cardiomyopathy in a 26-Year-Old Male

**DOI:** 10.7759/cureus.3011

**Published:** 2018-07-20

**Authors:** Jack Klenda, L K Teja Boppana, Mohinder R Vindhyal

**Affiliations:** 1 Internal Medicine, University of Kansas Medical School Wichita, Wichita, USA; 2 Internal Medicine, The University of the West Indies, Port of Spain, TTO; 3 Internal Medicine, University of Kansas School of Medicine - Wichita, Wichita, USA

**Keywords:** non-compaction, congestive heart failure

## Abstract

Left ventricular non-compaction (LVNC) is a genetic anomaly where the ventricular wall is replaced by thick ventricular trabeculations with deep intertrabecular recesses held together by a thin compacted layer. We present a case of a 26-year-old male who presented with dyspnea on exertion and edema in his legs for the last one week.

## Introduction

Left ventricular non-compaction cardiomyopathy (LVNC) is a rare congenital heart malformation. During embryogenesis, portions of myocardium fail to compact correctly. This leaves areas of the wall of the ventricles with a loosely compacted network that does not pump blood effectively. We present the case of a 26-year-old male who was admitted with LVNC, and the need to have a high index of clinical suspicion to suspect this diagnosis.

## Case presentation

**Figure 1:**
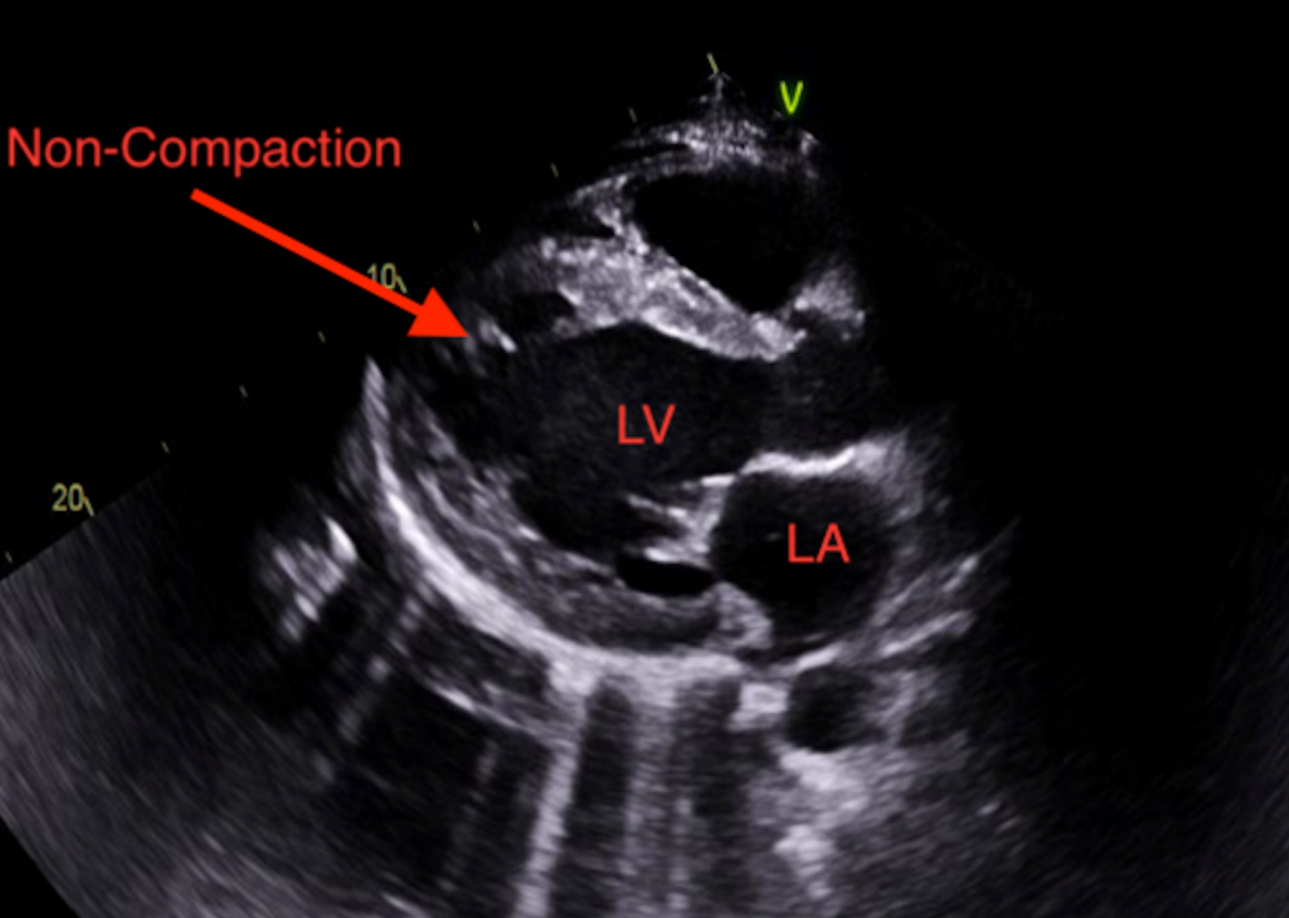
Parasternal long axis view showing LVNC (left ventricular non-compaction). LV: Left ventricle; LA: Left atrium.

**Figure 2:**
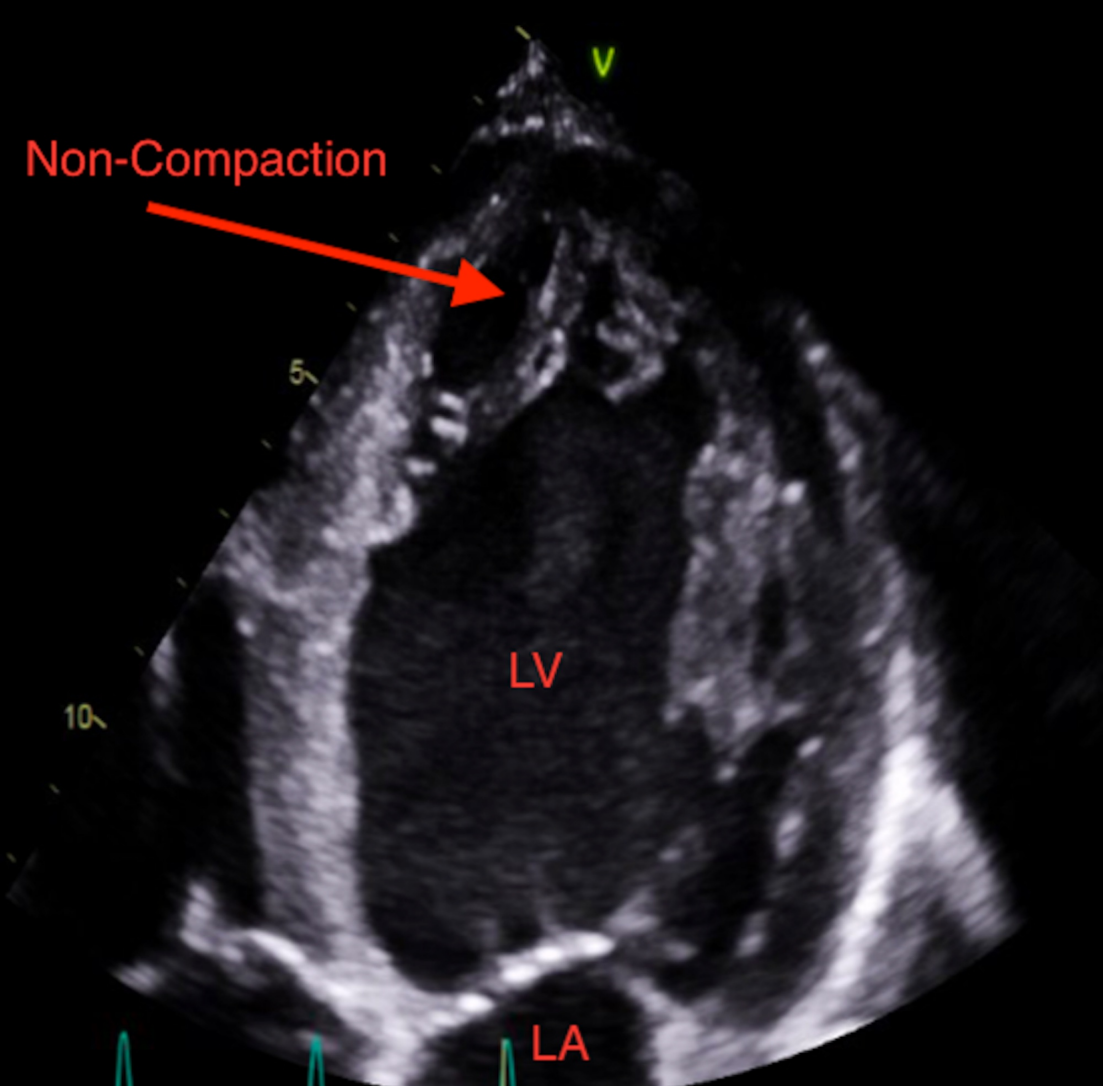
Apical 2 chamber view showing LVNC (left ventricular non-compaction). LV: Left ventricle; LA: Left atrium.

**Figure 3:**
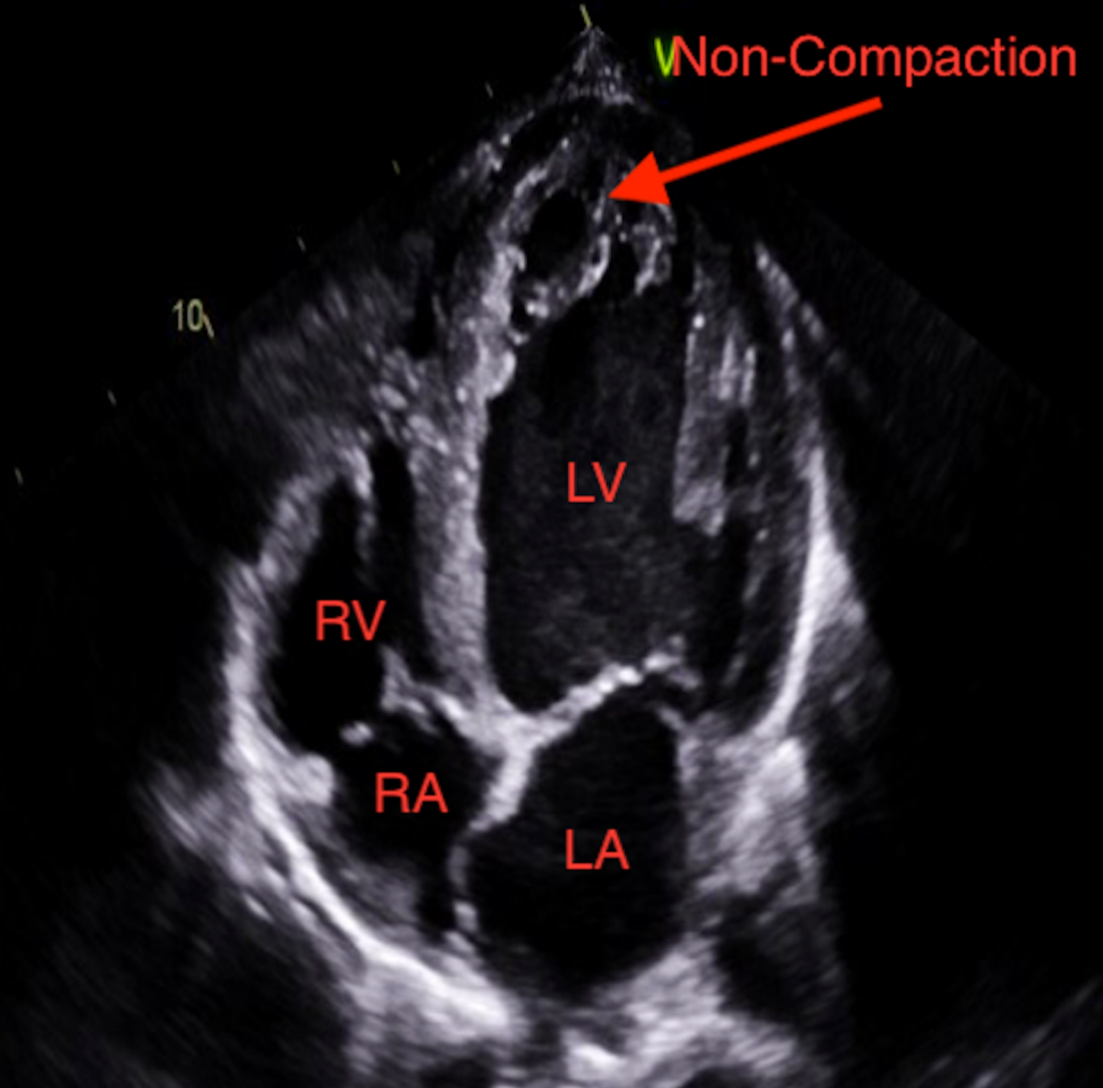
Apical 4 chamber view showing LVNC (left ventricular non-compaction). LV: Left ventricle; LA: Left atrium; RV: Right ventricle; RA: Right atrium.

A 26-year-old male patient with a past medical history of asthma and marijuana use presented to the emergency room with a one-week history of shortness of breath. The patient believed his shortness of breath was associated with his asthma and he did not seek help earlier. The patient reported having exertional dyspnea, orthopnea, paroxysmal nocturnal dyspnea, and blood-tinged sputum upon further questioning. He denied chest pain, palpitations, cough, fever or dizziness. The patient stated that he has moved recently from Washington State and is trying to establish care with a physician locally. The patient denies any surgeries, family history of medical conditions but endorses occasional alcohol use, daily marijuana use and no tobacco use. The patient lives with his sister and works as a cook at a local restaurant. He is sexually active with women, but not at the time of presentation. The patient states he has previously prescribed some medications but does not remember what they were, and reports that he has not been taking them. On physical exam, he appeared to be in moderate distress. His vital signs were abnormal with an elevated blood pressure of 182/110, increased pulse at 120/minute and increased respiratory rate at 24/minute with use of accessory respiratory muscles noted. The patient's body mass index was 23.4. Pulmonary examination revealed scattered wheezes in all lung fields and no crackles. The cardiovascular exam revealed a non-displaced point of maximal impulse. Cardiac auscultation was noted for tachycardia with no murmurs, rubs, or gallops. Examination of the head and neck showed dry mucous membranes, with minimal jugular venous distension. The gastrointestinal exam was largely normal with no hepatomegaly, splenomegaly, or tenderness to palpation. There was one plus edema noted on the extremities during the musculoskeletal exam. Laboratory investigations done showed that his creatinine at this time was 3.4 mg/dl. Further workup for his shortness of breath revealed elevated brain natriuretic peptide (BNP) at 2534 pg/mL. Echocardiogram followed, which revealed an ejection fraction (EF) of 10–15% and a diagnosis of LVNC was made with the help of image resolution, focusing in the apical region with a non-compaction to compaction ratio >2.3 as shown in Figures 1-3.

The EF was reassessed with the multiple-gated acquisition (MUGA) to be 27%. His acute kidney injury (AKI) was worked up and it resulted in a pre-renal etiology due to the cardio-renal syndrome. The patient was diuresed with furosemide 40 mg three times a day, which resulted in significant improvement of respiratory symptoms. However, renal function remained impaired throughout hospitalization. Creatinine peaked at 4.6 mg/dl but was trending down upon discharge. The patient was discharged on furosemide and carvedilol and was encouraged to follow fluid and salt-restricted diet. A life vest was acquired for home use, potentially as a bridge to automated implantable cardioverter-defibrillator (AICD).

## Discussion

Left ventricular non-compaction is a non-ischemic cardiomyopathy, where the endocardium and the myocardium do not develop normally during embryogenesis [1]. Engberding initially mentioned it in 1984 as isolated ‘sinusoids’ within the left ventricle [2]. The incidence of LVNC in infants is 0.81 per 100,000 infants/year, in children is 0.12 cases per 100,000 children/year and a prevalence of 0.014% in adults [3]. However, in adults presenting with heart failure, this rises to as much as 3% [4]. The etiology can be classified as non-familial (sporadic) or familial (sporadic) [3]. Non-familial forms are diagnosed when LVNC is proven absent in relatives [5] and are usually acquired, as in highly trained athletes, sickle cell anemia patients and in pregnancy [6-8]. Familial forms are confirmed by a thorough, three-generation family history for evaluation of genetic influence and screening of asymptomatic relatives of affected patients [9]. Numerous genes are associated with this condition and these include E101K mutation in α-cardiac actin (ACTC) gene, G4.5 gene on Xq28, cardiac-specific CSX gene and Fbkp1a/Notch pathway among others [9, 10]. There is also a genetic overlap with both dilated and hypertrophic cardiomyopathy [3]. Patients with LVNC (non-compaction) can present in both the pediatric or adult population. In the pediatric population, it may present with congenital abnormalities such as cyanotic congenital heart disease [11]. A study of 42 patients with LVNC by Yousef et al. in an adult population with a mean age of 48.7 found that the common symptoms and signs include dyspnea (50%), chest pain (19%) and palpitations (14%). Overall, the most common presentation remains heart failure (HF) followed by thromboembolism and arrhythmia (atrial and ventricular) [11]. The initial study of choice for the diagnosis of LVNC is the two-dimensional echocardiogram and this is a common test used for diagnosis [12]. Bennett and Freudenberger recommended using the Jenni criteria for the diagnosis of LVNC. Jenni criteria take into consideration both end-diastolic and end-systolic myocardial layer thickness. LVNC is diagnosed when non-compacted/compacted ratio >2.0 in end systole is found communicating with the intertrabecular space demonstrated by color Doppler with an absence of coexisting cardiac abnormalities and bi-layered myocardium with multiple, prominent trabeculations [13]. Cardiac magnetic resonance imaging (CMR) is another modality that is used for diagnosis. Low ejection fraction is not required for LVNC diagnosis. The high resolution of cardiac magnetic resonance helps differentiate non-compacted and compacted myocardium. It also allows for the imaging of the apex wall [9]. A study by Petersen et al. using CMR found that pathological non-compaction had a non-compaction to compaction ratio >2.3 in end-diastole and that the specificity and negative predictive values were both 99% [14]. Treatment for non-compaction focuses primarily on HF, embolic events, and arrhythmias. Treatment with ACE-Inhibitor and beta-blockers can be used to manage patients with HF and is similar to other causes of HF with reduced ejection fraction [1]. In addition, due to the familial association with LVNC, first degree relatives should be screened with an echocardiogram. Women with LVNC should be warned about future pregnancies to prevent worsening heart function [1].

## Conclusions

The diagnosis of LVNC can be made with echocardiogram once the clinician has elicited a thorough family history coupled with a high degree of clinical suspicion. Treatment includes managing heart failure, preventing sudden cardiac death with AICD and anticoagulation.

## References

[1] Weiford BC, Subbarao VD, Mulhern KM (2004). Noncompaction of the ventricular myocardium. Circulation.

[2] Engberding R, Bender F (1984). Identification of a rare congenital anomaly of the myocardium by two-dimensional echocardiography: persistence of isolated myocardial sinusoids. Am J Cardiol.

[3] Arbustini E, Weidemann F, Hall JL (2014). Left ventricular noncompaction: a distinct cardiomyopathy or a trait shared by different cardiac diseases?. J Am Coll Cardiol.

[4] Kovacevic-Preradovic T, Jenni R, Oechslin EN, Noll G, Seifert B, Attenhofer Jost CH (2009). Isolated left ventricular noncompaction as a cause for heart failure and heart transplantation: a single center experience. Cardiology.

[5] Zaragoza MV, Arbustini E, Narula J (2007). Compaction of the left ventricle: primary cardiomyopathy with an elusive genetic etiology. Curr Opinion Pediatrics.

[6] Gati S, Chandra N, Bennett RL (2013). Increased left ventricular trabeculation in highly trained athletes: do we need more stringent criteria for the diagnosis of left ventricular non-compaction in athletes?. Heart.

[7] Gati S, Papadakis M, Van Niekerk N, Reed M, Yeghen T, Sharma S (2013). Increased left ventricular trabeculation in individuals with sickle cell anemia: physiology or pathology?. Int J Cardiol.

[8] Gati S, Papadakis M, Papamichael ND (2014). Reversible de-novo left ventricular trabeculations in pregnant women: implications for the diagnosis of left ventricular noncompaction in low-risk populations. Circulation.

[9] Bennett CE, Freudenberger R (2016). The current approach to diagnosis and management of left ventricular noncompaction cardiomyopathy: review of the literature. Cardiol Res Pract.

[10] Monserrat L, Hermida-Prieto M, Fernandez X (2007). Mutation in the alpha-cardiac actin gene associated with apical hypertrophic cardiomyopathy, left ventricular non-compaction, and septal defects. Eur Heart J.

[11] Yousef ZR, Foley PWX, Khadjooi K, Chalil S, Sandman H, Mohammed NUH, Leyva F (2009). Left ventricular non-compaction: clinical features and cardiovascular magnetic resonance imaging. BMC Cardiovasc Disord.

[12] Shemisa K, Li J, Tam M, Barcena J (2013). Left ventricular noncompaction cardiomyopathy. Cardiovasc Diagn Ther.

[13] Jenni R, Oechslin E, Schneider J, Jost CA, Kaufmann PA (2001). Echocardiographic and pathoanatomical characteristics of isolated left ventricular non-compaction: a step towards classification as a distinct cardiomyopathy. Heart.

[14] Petersen SE, Selvanayagam JB, Wiesmann F (2005). Left ventricular non-compaction: insights from cardiovascular magnetic resonance imaging. J Am Coll Cardiol.

